# Cell–Electrode Models for Impedance Analysis of Epithelial and Endothelial Monolayers Cultured on Microelectrodes

**DOI:** 10.3390/s24134214

**Published:** 2024-06-28

**Authors:** Wei-Chih Chiu, Wei-Ling Chen, Yi-Ting Lai, Yu-Han Hung, Chun-Min Lo

**Affiliations:** 1 Department of Biomedical Engineering, National Yang Ming Chiao Tung University, Taipei 11221, Taiwan; alan1000224.y@nycu.edu.tw (W.-C.C.); bet1233.be11@nycu.edu.tw (W.-L.C.); 2 Research Center for Applied Sciences, Academia Sinica, Taipei 11529, Taiwan; ytlai@gate.sinica.edu.tw; 3 Department of Neurology, University of California, Irvine, CA 92697, USA

**Keywords:** electric cell–substrate impedance sensing (ECIS), cell–electrode model, impedance analysis, epithelial cells, endothelial cells

## Abstract

Electric cell–substrate impedance sensing has been used to measure transepithelial and transendothelial impedances of cultured cell layers and extract cell parameters such as junctional resistance, cell–substrate separation, and membrane capacitance. Previously, a three-path cell–electrode model comprising two transcellular pathways and one paracellular pathway was developed for the impedance analysis of MDCK cells. By ignoring the resistances of the lateral intercellular spaces, we develop a simplified three-path model for the impedance analysis of epithelial cells and solve the model equations in a closed form. The calculated impedance values obtained from this simplified cell–electrode model at frequencies ranging from 31.25 Hz to 100 kHz agree well with the experimental data obtained from MDCK and OVCA429 cells. We also describe how the change in each model-fitting parameter influences the electrical impedance spectra of MDCK cell layers. By assuming that the junctional resistance is much smaller than the specific impedance through the lateral cell membrane, the simplified three-path model reduces to a two-path model, which can be used for the impedance analysis of endothelial cells and other disk-shaped cells with low junctional resistances. The measured impedance spectra of HUVEC and HaCaT cell monolayers nearly coincide with the impedance data calculated from the two-path model.

## 1. Introduction

Electric cell–substrate impedance sensing (ECIS) has been developed as an instrumental method to electrically measure the interaction of adherent cells in culture with their substrate [1]. In this method, small gold electrodes serve as substrates for the cells, and the impedances of these electrodes to an alternating current (AC) signal are followed with time. The impedance increases when the cells attach and spread on the electrodes as their insulating plasma membranes restrict the current flow. These impedance values, measured at various AC frequencies, can be used to monitor cell attachment and spreading, cell morphology changes, and cell mobility with great sensitivity [2]. In addition, changes in cell morphology are observed as fluctuations in impedance, and these fluctuations can be numerically analyzed to give quantitative estimates of cellular micromotion [3].

One of the essential features of the ECIS method is the use of AC signals at various frequencies [2,4]. It is well known that AC techniques can provide information on the actual areas of folded cell membranes and, hence, membrane conductances [5]. The main basis for this concept is that a cell membrane can be electrically modeled as a resistor and a capacitor connected in parallel [6]. As a result, the amount of transcellular and paracellular currents varies while different frequencies of AC signals are applied. In addition, frequency-dependent changes in the measured impedance spectrum are related to cell morphology changes and cell–cell and cell–substrate interactions [4]. Previously, Giaever and Keese developed a cell–electrode model (GK model) to study cellular micromotion and analyze the impedance spectrum of fibroblasts measured by ECIS [3,7]. This model approximates a confluent cell layer as many connected circular disks on the sensing electrode. Electric currents flow radially in the space underneath the cell and then through basal and apical membranes (transcellular pathway) or around the cell through the intercellular space (paracellular pathway). This original two-path model includes three parameters for data fitting: junctional resistance between adjacent cells over a unit cell area (R_b_), a parameter relating to the resistance of the media-filled narrow channels beneath the cell (α), and transmembrane capacitance for a unit area (C_m_). By fitting the experimental data of confluent layers of WI-38 and WI-38 VA13 cells with the calculated values obtained from the GK model, model parameters, R_b_ and α, for both fibroblast types were successfully determined [3].

Electric cell–substrate impedance sensing, as the name implies, considers in detail the interaction of the adherent cells with the substrate they have attached to. A cleft, filled with culture media, separates the basal cell membrane from the electrode and thus generates a cleft resistance (R_cleft_) [8,9]. Unlike most equivalent circuit models used for cell-based impedance assays, the GK model includes the distributed effects of R_cleft_ underneath the cell layer. It is worth noting that R_cleft_ is distributed along with the distributed impedance of the basal cell membrane while the current flows radially in the cell–substrate cleft region and outward into the lateral intercellular space (LIS). This distributed effect is frequency-dependent and plays a significant role in the measured impedance spectrum. In ECIS, R_cleft_ can be defined as α^2^ = r_c_^2^(ρ/h), where r_c_ is the cell radius, ρ is the resistivity of the culture medium, and h is the average distance between the cells and the substratum (or the average distance of the cell–electrode cleft) [8].

**Figure 1:**
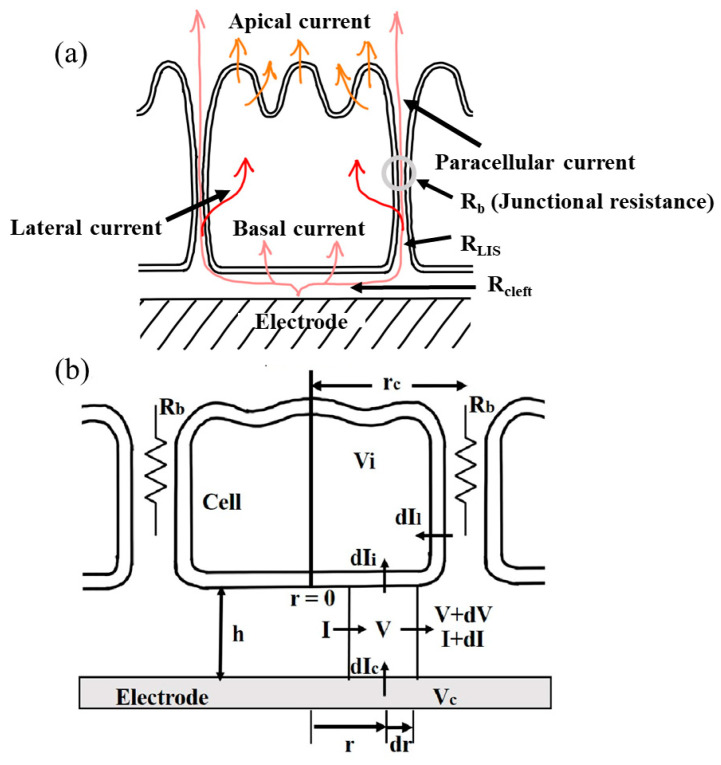
A schematic diagram of the simplified LGK model for cell layers cultured on a gold microelectrode. Cells are considered as disk-shaped. The side view (**a**) displays different current paths, junctional resistance (R_b_), LIS resistance (R_LIS_), and cleft resistance (R_cleft_). The side view (**b**) emphasizes the cell–substratum space and constructs Equations (1)–(4). The cell interior is assumed to be equipotential due to the relatively large cell volume and low intracellular resistivity.

Lo, Giaever, and Keese extended the GK model for the impedance analysis of epithelial cells [7,10]. This model (the LGK model), as illustrated in Figure 1, also describes cells as circular disks adhering to the sensing electrode. In addition to the two pathways defined in the GK model, the LGK model includes a third pathway for the electric currents to flow along the LIS. In through the lateral membrane and out through the apical membrane, this pathway is transcellular and more applicable to most epithelial cells [5,11]. This is because epithelial cells have higher junctional resistances than fibroblasts or endothelial cells, and the membrane impedance decreases at high frequencies. This third pathway is necessary for the model calculation of epithelial cells, especially when high-frequency AC signals are used [5]. In the LGK model, paracellular resistance consists of a tight junction resistance (R_b_) and LIS resistance (R_LIS_). Similar to the distributed effects of R_cleft_ and the impedance of the basal cell membrane, distributed R_LIS_ and the distributed impedance of the lateral cell membrane are also considered in the model. The LGK model includes six adjustable parameters: R_b_, α, C_a_, (apical membrane capacitance per unit area), C_b_, (basal membrane capacitance per unit area), C*_l_* (lateral membrane capacitance per unit area), and R*_l_* (the resistance of the LIS per unit length) [10]. By comparing the calculated impedance spectra of the cell-covered electrodes with those measured for confluent layers of MDCK epithelial cells, the LGK model has been demonstrated to fit the experimental data precisely [10].

Although the LGK model is useful for the impedance analysis of epithelial cells, data fitting is challenging if too many fitting parameters are used. Given the purpose of easy use of the cell–electrode model, it is important to limit the number of model parameters to only necessary ones. Among the six parameters in the LGK model, we find it most difficult to adjust the parameter R_l_ (R_LIS_ per unit length) for data fitting. Here, we aim to simplify the LGK model by disregarding the contribution of R_l_ and reducing the fitting parameters from six to five. If R_LIS_ is much lower than R_b_, R_LIS_ can be considered negligible (i.e., R_LIS_ << R_b_), and the distributed effect of the lateral membrane is ignored; therefore, the LGK model can be simplified to only five fitting parameters. The feasibility of LGK model simplification is checked numerically as follows: In the simplest estimation, the resistance of the LIS per unit area can be calculated as R_LIS_ = [ρ*l*/(2πr_c_)(0.5*w*)](πr_c_^2^), where ρ, *l*, *w*, and r_c_, are the resistivity of culture medium, LIS length, LIS width, and cell radius respectively [5]. For confluent MDCK cells, the LIS is approximately 0.25~1.00 μm wide (*w*) and 6 μm long (*l*) [12], and the cell radius (r_c_) is 7 μm [13]. With ρ = 60 Ω·cm, the R_LIS_ ranges from 0.25 to 1.00 Ω·cm^2^ for an LIS width of 1.00 to 0.25 μm. Yet, the junctional resistance per unit area of MDCK cells (R_b_) is around 60~100 Ω·cm^2^ (relevant results from this work, which will be described in detail below), which is around 100 times larger than the R_LIS_.

In this study, we develop a simplified LGK model by ignoring R_LIS_ and treating lateral membranes as lumped impedances. We solve the second-order differential equation in a closed form, and the total impedance only depends on five parameters: R_b_, α, C_a_, C_b_, and C_l_. We also evaluate how each model parameter affects the impedance spectra of the cell-covered electrodes and apply this model to analyze the experimental data obtained from MDCK and OVCA429 cells. The third pathway in the LGK model can be ignored if the junctional resistance is smaller than the specific impedance of the lateral cell membrane. Based on this assumption, the simplified LGK model is further reduced to a modified GK model, which contains only four fitting parameters: R_b_, α, C_a_, and C_b_. The measured impedance spectra of HUVEC and HaCaT cells are analyzed using the modified GK model and compared with the relevant results obtained from the simplified LGK model. The overall calculated results from the simplified LGK model or modified GK model for all four cell types agree well with the measured impedance spectra.

## 2. Materials and Methods

### 2.1. Cell Culture

MDCK epithelial cells (kindly provided by Dr. Yeh-shiu Chu, National Yang Ming Chiao Tung University, Taiwan) were cultured in DMEM with 1.0 g/L glucose (Gibco, Grand Island, NY, USA) supplemented with 10% (*v*/*v*) fetal bovine serum (Gibco), 100 U/mL penicillin (Gibco), 100 μg/mL streptomycin (Gibco), and 250 ng/mL amphotericin B (Gibco). OVCA429 ovarian cancer cells were kindly provided by Dr. Samuel C. Mok at MD Anderson Cancer Center, Houston, TX, USA. These cells were grown in M199 and MCDB 105 (1:1) (Sigma, St. Louis, MO, USA) supplemented with 10% fetal calf serum (Sigma), 2 mM L-glutamine, 100 U/mL penicillin (Gibco), and 100 μg/mL streptomycin (Gibco).

Human umbilical vein endothelial cells (HUVECs, Clonetics Corp., San Diego, CA, USA) were cultured in endothelial cell growth medium (EGM; Clonetics Corp.) which was supplemented with 10 ng/mL human recombinant epidermal growth factor, 1 μg/mL hydrocortisone, 50 μg/mL gentamicin, 50 ng/mL amphotericin B, 12 μg/mL bovine brain extract, and 2% fetal bovine serum (Gibco). HUVECs passaged fewer than six times were used in experiments. Human keratinocytes (HaCaT cells, provided by Dr. Jehng-Kang Wang, National Defense Medical Center, Taipei, Taiwan) were cultured in DMEM with 4.5 g/L glucose (Gibco) and supplemented with 10% fetal bovine serum (Gibco), 100 U/mL penicillin, 100 μg/mL streptomycin (Gibco), and 1% L-glutamine solution (Corning, New York, NY, USA). All cell lines were cultured under 5% CO_2_ and a 37 °C high-humidity atmosphere. Cells were subcultured with a standard trypsinization procedure when they were 70% confluent, and the medium was changed every two days after that.

### 2.2. Impedance Measurements Using ECIS

Electrode arrays (8W1E), an ECIS Zθ instrument, and the acquired software (Version 2.215) for ECIS measurement were obtained from Applied Biophysics (Troy, NY, USA). A lock-in amplifier was used to measure in- and out-of-phase voltages through the sensing electrode at various frequencies. These voltage data were then mathematically converted to resistance and capacitance values considering the cell–electrode system as a series RC circuit. It is noteworthy that this conversion is a standard AC circuit analysis. Treating the cell–electrode system as a parallel RC circuit can obtain equally good results. However, since all components in a series circuit share the same current, applying Ohm’s law to determine resistance and capacitive reactance from a series RC circuit is more straightforward than a parallel RC circuit. For ECIS studies, cells were taken from slightly subconfluent cultures 48 h after passage, and a monodisperse cell suspension was prepared using standard tissue culture techniques with trypsin/EDTA. These suspensions were equilibrated at incubator conditions before addition to the electrode-containing wells. Confluent cell layers were formed using 10^5^ cell/cm^2^ inoculation density.

We monitored cell attachment and spreading using multiple-frequency time series (MFT) measurements with 11 pre-defined frequencies ranging from 62.5 Hz to 64 kHz. These frequencies are 62.5 Hz, 125 Hz, 250 Hz, 500 Hz, 1 kHz, 2 kHz, 4 kHz, 8 kHz, 16 kHz, 32 kHz, and 64 kHz. We also applied a frequency scan measurement with an impedance calculation from the cell–electrode model to determine the morphological parameters of each cell line. In this method, we measured the impedances of the cell–electrode system at 25 different frequencies, ranging from 31.25 Hz to 100 kHz. Each frequency (except the first and last) was generated by multiplying the previous one by 2^1/2^. Because the lock-in amplifier generates single-frequency sine waves with an operating range of 1 mHz to 102 kHz, instead of 128 kHz, we use 100 kHz as the highest frequency for the frequency scan measurement. The calculated values obtained from different cell–electrode models (Z_cal_) were fitted with these measured impedance data (Z_exp_), including 25 resistance and 25 capacitance reactance points. By using the method of non-linear least squares, we calculated the sum of the squares of the percentage errors (Z_cal_ − Z_exp_)/Z_exp_, and refined the model parameters until the minimum sum is attained [14].

### 2.3. Model Derivation

#### 2.3.1. The Simplified LGK Model

The assumptions used for deriving the simplified LGK model are similar to the LGK model assumptions, which have been previously described [3]. From Figure 1 and Kirchhoff’s Law for AC circuits, we get

(1) $$-\frac{dV}{dr}=\frac{\rho }{h2\pi r}I,$$

(2) $$V_{c}-V=\frac{Z_{n}}{2\pi rdr}dI_{c},$$

(3) $$V-V_{i}=\frac{Z_{b}}{2\pi rdr}dI_{i},$$

(4) $$dI=dI_{c}-dI_{i}.$$

Equations (1)–(4) can be combined to yield the following differential equation:

(5) $$\frac{d^{2}V}{dr^{2}}+\frac{1}{r}\frac{dV}{dr}-\gamma^{2}V+\beta =0,$$

where

(6) $$\gamma^{2}=\frac{\rho }{h}\left(\frac{1}{Z_{n}}+\frac{1}{Z_{b}}\right)$$

and

(7) $$\beta =\frac{\rho }{h}\left(\frac{V_{c}}{Z_{n}}+\frac{V_{i}}{Z_{b}}\right).$$

The general solution of Equation (5) is

(8) $$V=AI_{0}\left(\gamma r\right)+BK_{0}\left(\gamma r\right)+\frac{\beta }{\gamma^{2}},$$

where *I_0_*(*γr*) and *K_0_*(*γr*) are modified Bessel functions of the first and second kind, respectively. Since *K_0_*(*γr*) diverges as r goes to zero and the domain of the general solution is [0, *r_c_*], the coefficient B is zero. The general solution of Equation (5) becomes

(9) $$V=AI_{0}\left(\gamma r\right)+\frac{\beta }{\gamma^{2}}.$$

We assume that the electrical potential inside the cell, V_i_, is a constant. For the transcellular current through the apical cell surface, the equation related to the transcellular current can be expressed as

(10) $$V_{i}=\frac{Z_{a}}{\pi r_{c}^{2}}I_{i}.$$

Combining Equations (1) and (9), electrical current through the space between the cell layer and electrode can be expressed as

(11) $$I=-\frac{-2\pi rhA\gamma }{\rho }I_{1}\left(\gamma r\right).$$

In the simplified LGK model, we assume that the resistance of LIS per unit area (R_LIS_) is much lower than the junctional resistance per unit area (R_b_) and can be ignored in the model derivation. We also assume that the distributed effect of the lateral membrane can be ignored. Therefore, the equation related to the paracellular current passing through the lateral cell membrane (I*_l_*) can be expressed as

(12) $$V\left(r=r_{c}\right)-V_{i}=\frac{Z_{L}}{2\pi r_{c}l}I_{l}.$$

Two boundary conditions for determining the two constants, A and V_i_, are as follows:

(13) $$V\left(r=r_{c}\right)=\left[I\left(r=r_{c}\right)-I_{l}\right]\times R_{b}^{*}$$

and

(14) $$I_{c}=I_{i}+I\left(r=r_{c}\right)-I_{l}.$$

We obtain the following matrix equation from the boundary conditions, Equations (13) and (14).

(15) $$\left[\begin{array}{cc} \left(1+\frac{G}{Z_{l}}\right)I_{0}\left({\gamma r}_{c}\right)+\left(\frac{2R_{b}h\gamma }{r_{c}\rho }\right)I_{1}\left({\gamma r}_{c}\right) & \left(1+\frac{G}{Z_{l}}\right)\frac{Z_{n}}{Z_{n}+Z_{b}}-\frac{G}{Z_{l}}\\ \frac{I_{0}\left({\gamma r}_{c}\right)}{R_{b}}+\frac{{2I}_{1}\left({\gamma r}_{c}\right)}{Z_{n}{\gamma r}_{c}} & \frac{1}{Z_{a}}+\frac{\left(1+\frac{Z_{n}}{R_{b}}\right)}{Z_{n}+Z_{b}} \end{array}\right]\left[\begin{array}{c} A\\ V_{i} \end{array}\right]=\left[\begin{array}{c} -\left(1+\frac{G}{Z_{l}}\right)\frac{Z_{b}V_{c}}{Z_{n}+Z_{b}}\\ \left(1-\frac{Z_{b}}{R_{b}}\right)\frac{V_{c}}{Z_{n}+Z_{b}} \end{array}\right],$$

where

(16) $${\gamma r}_{c}=r_{c}\sqrt{\frac{\rho }{h}\left(\frac{1}{Z_{n}}+\frac{1}{Z_{b}}\right)}=\alpha \sqrt{\left(\frac{1}{Z_{n}}+\frac{1}{Z_{b}}\right)}.$$

Using matrix algebra, we determine the numerical constants A and V_i_ by solving the matrix equation. The total current from the area of the single cell, I_ct_, can be found by integrating Equation (2):

(17) $$I_{ct}=\int_{0}^{r_{c}}\frac{2\pi r}{Z_{n}}\left(V_{c}-V\right)dr=\frac{-2\pi r_{c}^{2}A}{Z_{n}\gamma r_{c}}I_{1}\left(\gamma r_{c}\right)+\frac{\pi r_{c}^{2}}{Z_{n}+Z_{b}}\left(V_{c}-V_{i}\right).$$

For the simplified LGK Model, the exact solution of the specific impedance of the cell-covered electrode, Z_c_, is:

(18) $$\frac{1}{Z_{c}}=\frac{I_{ct}}{\pi r_{c}^{2}V_{c}}=\frac{1}{Z_{n}+Z_{b}}\left\{1+\frac{\frac{2Z_{b}\left(1\;+\;\frac{G}{Z_{l}}\right)}{Z_{n}}}{Den1}-\frac{\left\{\left(1-\frac{Z_{b}}{R_{b}}\right)+\frac{Z_{b}\left(1\;+\;\frac{G}{Z_{l}}\right)\;\times \;Num1}{Den1}\right\}\left\{1+\frac{2\left(\frac{G}{Z_{l}}Z_{b}\;-\;Z_{n}\right)}{Z_{n}\;\times \;Den1}\right\}}{\frac{Z_{a}\;+\;Z_{n}\;+\;Z_{b}}{Z_{a}}+\frac{Z_{n}}{R_{b}}+\frac{\left(\frac{G}{Z_{l}}Z_{b}\;-\;Z_{n}\right)\;\times \;Num1}{Den1}}\right\},$$

where

(19) $$Den1=\gamma r_{c}\frac{I_{0}\left(\gamma r_{c}\right)}{I_{1}\left(\gamma r_{c}\right)}\left(1+\frac{G}{Z_{l}}\right)+2R_{b}\frac{Z_{n}+Z_{b}}{Z_{n}Z_{b}},$$

(20) $$Num1=\frac{2}{Z_{n}}+\frac{\gamma r_{c}}{R_{b}}\frac{I_{0}\left(\gamma r_{c}\right)}{I_{1}\left(\gamma r_{c}\right)},$$

(21) $$G=2\pi r_{c}lR_{b}^{*}=\frac{2lR_{b}}{r_{c}},$$

(22) $$Z_{n}=S\left(R_{n}+\frac{1}{i2\pi fC_{n}}\right),$$

(23) $$Z_{a}={\left(\frac{1}{R_{m}}+i2\pi fC_{a}\right)}^{-1},$$

(24) $$Z_{b}={\left(\frac{1}{R_{m}}+i2\pi fC_{b}\right)}^{-1},$$

and

(25) $$Z_{l}={\left(\frac{1}{R_{m}}+i2\pi fC_{l}\right)}^{-1}.$$

Here, *S* is the sensing electrode area of 5 × 10^−4^ cm^2^. We define the specific impedance of the cell-free electrode, Z_n_, as a resistance (R_n_) and a capacitance (C_n_) connected in series as shown in Equation (22). R_n_ and C_n_ are obtained respectively from in-phase and out-of-phase data measured by the lock-in amplifier at different frequencies. We assume that the specific impedances of the apical, basal, and lateral membranes, Z_a_, Z_b_, and Z*_l_*, can be calculated as a resistor and a capacitor in parallel, as shown in Equations (23)–(25). We also assume that the specific resistance of the cell membrane (R_m_) is 10^3^ Ω·cm^2^, commonly given for biological membranes [15]. The model equation (Equation (18)) calculates the specific impedance of the cell-covered electrode, Z_c_, with the measured Z_n_ and five model parameters, specifically R_b_, α, C_a_, C_b_, and C*_l_*. Both resistance and reactance components of the calculated Z_c_ fit the measured Z_c_ at 25 different frequencies, allowing the determination of the five model parameters.

#### 2.3.2. The Modified GK Model

Similar to the simplified LGK model, we use Equations (1)–(11) to derive the modified GK model. As shown by most fibroblasts and endothelial cells, the junctional resistance between adjacent cells, R_b_, is negligible compared with the specific impedance through the lateral cell membrane, Z*_l_*. In this case, the third pathway for the electric currents to flow along the LIS and through the lateral membrane no longer exists. Two boundary conditions, Equations (13) and (14), for determining A and V_i_ can be rewritten as:

(26) $$V\left(r=r_{c}\right)=I\left(r=r_{c}\right)\times R_{b}^{*}$$

and

(27) $$I_{c}=I_{i}+I\left(r=r_{c}\right).$$

We then obtain the following matrix equation from boundary conditions, Equations (26) and (27).

(28) $$\left[\begin{array}{cc} I_{0}\left(\gamma r_{c}\right)+\left(\frac{2R_{b}h\gamma }{r_{c}\rho }\right)I_{1}\left(\gamma r_{c}\right) & \frac{Z_{n}}{Z_{n}+Z_{b}}\\ \frac{I_{0}\left(\gamma r_{c}\right)}{R_{b}}+\frac{2I_{1}\left(\gamma r_{c}\right)}{Z_{n}\gamma r_{c}} & \frac{1}{Z_{a}}+\frac{\left(1+\frac{Z_{n}}{R_{b}}\right)}{Z_{n}+Z_{b}} \end{array}\right]\left[\begin{array}{c} A\\ V_{i} \end{array}\right]=\left[\begin{array}{c} -\frac{Z_{b}V_{c}}{Z_{n}+Z_{b}}\\ \left(1-\frac{Z_{b}}{R_{b}}\right)\frac{V_{c}}{Z_{n}+Z_{b}} \end{array}\right]$$

For the modified GK Model, the exact solution of the specific impedance of the cell-covered electrode, Z_c_, is:

(29) $$\frac{1}{Z_{c}}=\frac{I_{ct}}{\pi r_{c}^{2}V_{c}}=\frac{1}{Z_{n}+Z_{b}}\left\{1+\frac{\frac{2Z_{b}}{Z_{n}}}{Den2}-\frac{{\left(\frac{2}{Den2}-1\right)}^{2}}{\frac{Z_{a}\;+\;Z_{n}\;+\;Z_{b}}{Z_{a}}\;+\;\frac{\frac{2Z_{n}}{Z_{b}}}{Den2}}\right\},$$

where

(30) $$Den2=\gamma r_{c}\frac{I_{0}\left(\gamma r_{c}\right)}{I_{1}\left(\gamma r_{c}\right)}+2R_{b}\frac{Z_{n}+Z_{b}}{Z_{n}Z_{b}}.$$

As expected, Equations (29) and (30) can be easily obtained by plugging (*G*/*Z_l_*) = 0 into Equations (18) and (19). The model equation (Equation (29)) calculates the specific impedance of the cell-covered electrode, Z_c_, with the measured Z_n_ and four model parameters, specifically R_b_, α, C_a_, and C_b_.

## 3. Results

### 3.1. Measurement of MDCK Cell Attachment and Spreading

**Figure 2:**
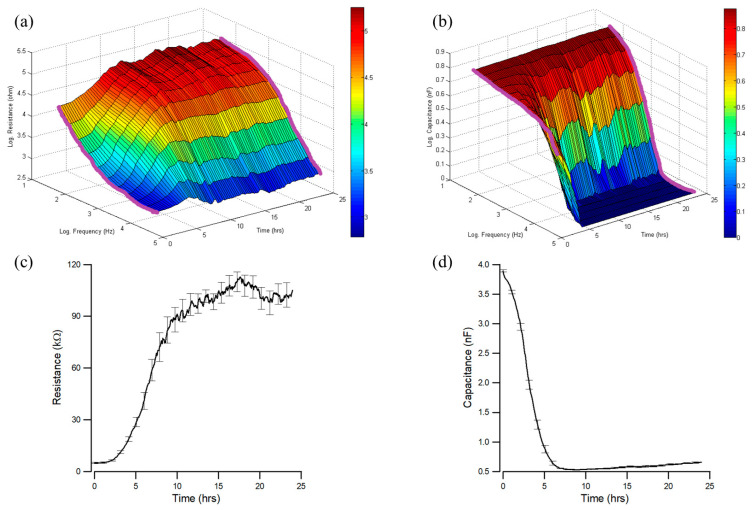
Multiple-frequency time series (MFT) measurements of MDCK cell attachment and spreading. Three-dimensional plots (**a**,**b**) are the log of measured resistance and capacitance as a function of log frequency and time. Bold purple curves indicate the impedance spectra at time 0 and 24 h after seeding cells. Plots (**c**,**d**) are the time series resistances and capacitances measured at 500 Hz and 64 kHz, respectively. Data were averaged from eight electrode wells and presented as mean ± standard error.

To monitor MDCK cell attachment and spreading, we inoculated cells into electrode wells at a 10^5^ cells/cm^2^ cell density. Electrical impedances of the cell-covered electrodes were followed at various frequencies for 24 h. Three-dimensional illustrations of the changes in measured resistance and capacitance as a function of frequency (spectra) and time are shown in Figure 2a,b. Both resistance and capacitance spectra dramatically change in the first 10 h after cell seeding. In addition, resistance and capacitance time series traced at different frequencies vary in various ways. For example, the measured resistances (Figure 2a) at low frequencies (≤1 kHz) increase quickly in the first 10 h and then slowly reach their final values. However, the measured resistances at high frequencies (>1 kHz) increase in the first 10 h and then drop slightly to their final values. Looking at the measured capacitance time series in the first 10 h (Figure 2b), they significantly decrease at high frequencies (≥4 kHz) but only gradually decrease at low frequencies (<4 kHz). 

We have defined the optimal detection frequency as the frequency at which the seeded cells contribute the maximum relative change in the measured resistance or capacitance [16]. For MDCK cell attachment and spreading monitored by MFT runs, the optimal detection frequencies for tracing resistance and capacitance changes are 500 Hz and 64 kHz [16]. Figure 2c,d show typical 24 h resistance and capacitance time series data measured at 500 Hz and 64 kHz. Specifically, the measured resistance increases from 5 kΩ to 90 kΩ and then 105 kΩ at 0, 10 h, and 24 h after cell inoculation (Figure 2c). At the same time, the measured capacitance decreases from 3.9 nF to 0.54 nF and then 0.66 nF, as shown in Figure 2d, indicating the formation of a confluent MDCK monolayer [2]. Resistance or capacitance tracings fluctuate even after the MDCK cells are confluent. These fluctuations are due to cellular micromotions caused by the rearrangement of cell–cell and cell–substrate adhesion sites.

### 3.2. Parameter Analysis of the Simplified LGK Model

**Figure 3:**
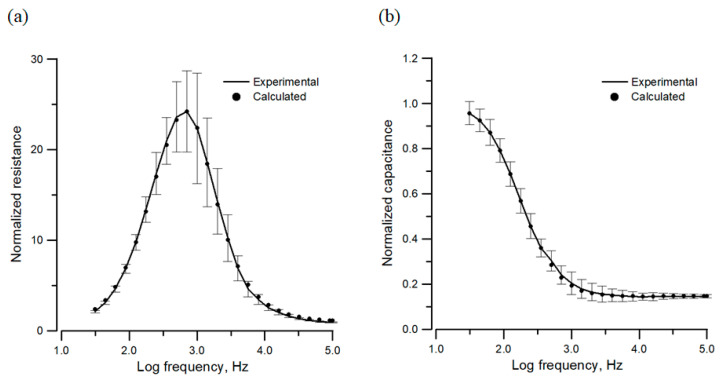
(**a**) Normalized resistance and (**b**) normalized capacitance spectra of an electrode covered with confluent MDCK cells. The curves are obtained from measured resistance and capacitance values at 25 different frequencies by dividing them by the corresponding quantities of the same electrode without cells. The points are calculated values from the simplified LGK model using Equations (18)–(20). Measured values are means ± standard error; *n* = 14.

Generally, we use frequency scans before and after cells attach and spread on the electrode to obtain impedances as a function of frequency for both a cell-free electrode and the same electrode confluent with cells. The data curves are similar to the bold purple curves shown in Figure 2a,b, which are the resistance and capacitance spectra at time zero and 24 h after seeding cells. Figure 3 displays the normalized resistance and capacitance spectra of MDCK cells, which are obtained by dividing the resistance and capacitance spectra of a cell-covered electrode by the corresponding values of the same electrode without cells. We used the simplified LGK model equations (Equations (18)–(20)) and the impedance spectrum of the cell-free electrode to calculate and fit both the resistance and capacitance spectra at 25 different frequencies. The best fitting values of the model parameters of MDCK cells, R_b_, α, C_a_, C_b_, and C*_l_*, are 88 Ω·cm^2^, 27 Ω^1/2^·cm, 4.5 μF/cm^2^, 2.0 μF/cm^2^, 1.6 μF/cm^2^, respectively. We estimate the cleft resistance (R_cleft_ = α^2^), and the average value of the R_cleft_ is around 729 Ω·cm^2^. This resistance is surprisingly large, implying the ventral surfaces of MDCK cells are closely in contact with the sensing electrode.

**Figure 4:**
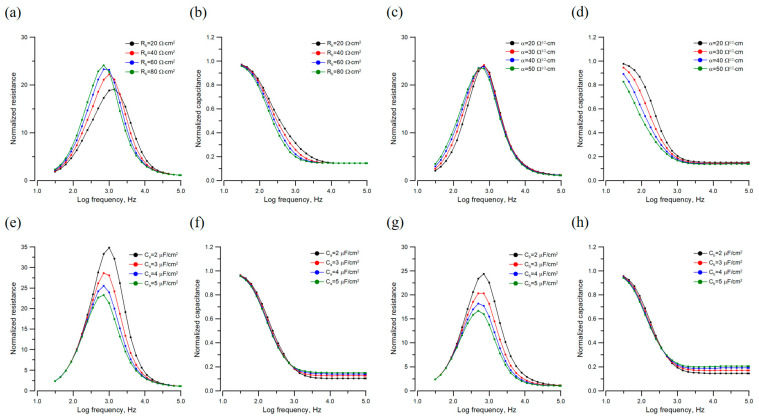
Normalized resistance (**a**,**c**,**e**,**g**) and normalized capacitance (**b**,**d**,**f**,**h**) spectra are calculated from the simplified LGK model using Equations (18)–(20). Here we use R_b_ = 88 Ω·cm^2^, α = 27 Ω^1/2^·cm, C_a_ = 4.5 μF/cm^2^, C_b_ = 2.0 μF/cm^2^, and C*_l_* = 1.6 μF/cm^2^ as the basis parameters for model calculations. These values are close to the fitted results of MDCK cells. We change only one parameter in the model calculation, and the parameter values used for model calculation are indicated in each figure.

Knowing how changes in each model parameter affect the normalized impedance spectrum is crucial to getting a good model fitting. Figure 4a,b show the normalized resistance and capacitance spectra for different values of R_b_: 20, 40, 60, and 80 Ω·cm^2^. When R_b_ increases, the peak of the normalized resistance spectrum (Figure 4a) shifts leftward and upward. As a result, the values of the normalized resistance increase on the low-frequency side and decrease on the high-frequency side. Figure 4c,d show another normalized resistance and capacitance spectra for different values of α: 20, 30, 40, and 50 Ω^1/2^·cm. When α increases, the left side of the normalized resistance curve (Figure 4c) moves leftward, whereas the peaks of all the curves are almost at the same height. As for normalized capacitance spectra shown in Figure 4b,d, capacitance values at different frequency ranges decrease as R_b_ or α increases. Finally, as shown in Figure 4e,g, when C_a_ or C_b_ increases from 2 μF/cm^2^ to 5 μF/cm^2^, the right side of the normalized resistance curve moves leftward and downward. In general, while changes in C_a_ or C_b_ give similar effects to the normalized resistance and capacitance spectra, parameter C_a_ has more impact than C_b_ on the calculated spectrum. Notably, both normalized resistance and capacitance spectra change slightly when C*_l_* takes on the values of 1.0, 1.5, 2.0, and 2.5 μF/cm^2^.

### 3.3. Cell Parameter Values Obtained from Different Cell–Electrode Models

We also applied the simplified LGK model to analyze impedance spectra obtained from OVCA429 cells; the results are shown in Table 1. Our results show that both R_b_ and α values of OVCA429 cell layers obtained from the simplified LGK model are higher than those of MDCK cell layers. The values of the other three parameters, Ca, Cb, and Cl, are similar for these cell types. We compare the results obtained from different cell–electrode models by fitting the same impedance spectra using LGK and modified GK models (Table 1). For MDCK and OVCA429 cells, all the parameter values fitted from the simplified LGK model are closer to those from the LGK model. On the other hand, the values of parameters R_b_ and C_b_ from the modified GK model are higher than those obtained from simplified LGK and LGK models. In addition, the values of parameter α and C_a_ from the modified GK model are smaller than those obtained from simplified LGK and LGK models.

We carried out several frequency scan measurements of HaCaT and HUVEC cell layers. These impedance spectra data were analyzed using simplified LGK and modified GK models, and the results are shown in Table 2. Interestingly, the junctional resistances (Rb) from the simplified LGK model for HaCaT and HUVECs are 1.4 Ω·cm^2^ and 0.6 Ω·cm^2,^ respectively, very close to the values obtained from the modified GK model, 1.6 Ω·cm^2^ and 0.7 Ω·cm^2^. Moreover, the average values of the parameter α from the simplified LGK model for HaCaT and HUVECs are 3.6 Ω^1/2^·cm and 5.0 Ω^1/2^·cm, respectively, also close to the values obtained from the modified GK model, 3.4 Ω^1/2^·cm and 4.9 Ω^1/2^·cm. Our results, shown in Table 1 and Table 2, indicate that compared with epithelial cell layers such as MDCK and OVCA429, the junctional resistances (R_b_) and cleft resistances (α^2^) of HaCaT and HUVEC are very small. Therefore, the third current pathway is unnecessary for HaCaT and HUVEC cells.

## 4. Discussion

We studied cell attachment and spreading on sensing electrodes using ECIS MFT measurement, which is useful for investigating in vitro cell behaviors in real time. Figure 2a,b are examples of three-dimensional plots to demonstrate early dramatic changes in resistance and capacitance spectra (t < 10 h) caused by MDCK cells. The peak value of the normalized resistance spectra, as shown in Figure 3a, represents the maximum relative change in resistance (R_cell-covered_/R_cell-free_), and the peak position is at 707 Hz. Among the 11 frequencies in the MFT runs, 500 Hz is the closest to 707 Hz and can be used to trace time series resistance changes for MDCK cells. In Figure 2c, the early resistance increase (t < 10 h) is mainly due to the cell attachment and spreading. The later resistance increase (t > 10 h) is primarily due to the formation of a barrier function [2]. Time series capacitance curves traced at 64 kHz (Figure 2d) also reveal a confluent MDCK cell layer formation within 10 h after cell seeding. These results are consistent with previous reports [2,16]. 

Cell–electrode models provide theoretical bases for impedance analysis of cell layers measured by ECIS regarding the morphological parameters and cell characteristics in tissue culture [3,10,14,17]. Figure 3 demonstrates the ability of the simplified LGK model to fit the measured impedance spectra of confluent MDCK cell layers precisely. Our analytical approach in this paper provides insight into how changes in each cell parameter influence measured impedance spectra. As a result of these modeling studies, one crucial fact is shown in Figure 4a, where the normalized resistance values of MDCK cells increase in the lower frequency region (<1 kHz) but decrease in the higher frequency region (>1 kHz) as the parameter R_b_ increases. This information explains why the measured time series resistances at high frequencies increase in the first 10 h and gradually drop after the barrier function is formed (Figure 2a). In contrast, using resistance tracing at 500 Hz (Figure 2c), we successfully monitor the formation of a barrier function. Similarly, the analysis shown in Figure 4b explains why at high frequencies (≥8 kHz), the measured time series capacitances do not change much even when the barrier function is formed (Figure 2b). Therefore, capacitance tracings at high frequencies such as 64 kHz (Figure 2d) are usually used for monitoring cell coverage on the sensing electrode, implying applications for ECIS wound healing migration assays. 

In this study, we have developed simplified LGK and modified GK models and compared the calculated impedance spectra to those measured for confluent layers of MDCK, OVCA429, HaCaT, and HUVEC cell layers. These two models have been demonstrated to fit the experimental data precisely, and the results are shown in Table 1 and Table 2. For MDCK and OVCA429 cells, all the parameter values fitted from the simplified LGK model are close to those from the LGK model. This result agrees with our assumption that if R_LIS_ is much lower than R_b_, then R_LIS_ can be considered negligible in the model derivation, and the distributed effect of the lateral membrane can also be ignored. The simplified LGK model is helpful for the impedance analysis of epithelial cells like MDCK and OVCA429.

The major difference between the modified GK model and the simplified LGK model is the consideration of the third pathway, where the currents pass along the LIS, in through the lateral membrane, and out through the apical membrane. As a result, the junctional resistances (R_b_) of MDCK and OVCA429 cells fitted by the modified GK model (two-path model) are overestimated, while the α parameters are somewhat underestimated. Since the modified GK model does not consider the third path, the total current passing through the basal cell membrane is the same as that through the apical cell membrane. Without considering the third-path currents, applying the modified GK model to epithelial cells would overestimate the currents through the basal cell membrane and underestimate the currents through the apical cell membrane. Since capacitive reactance is inversely proportional to the capacitance, this problem causes a slight underestimation of the C_a_ and significantly overestimates the C_b_ in the modified GK model (Table 1). For example, C_b_ values of MDCK and OVCA429 cells are 2.8 μF/cm^2^ and 2.6 μF/cm^2^ from the simplified LGK model and 3.6 μF/cm^2^ and 2.9 μF/cm^2^ from the modified GK model. Since the third current path is not considered in the modified GK model, the large C_b_ value from the modified GK model represents the capacitance of the basolateral membrane rather than the basal membrane only. In contrast, considering the third current path for HaCaT and HUVEC cells causes an underestimation of the C_b_ value. As shown in Table 2, C_b_ values of HaCaT and HUVEC cells are 2.1 μF/cm^2^ and 2.8 μF/cm^2^ from the modified GK model and 1.6 μF/cm^2^ and 2.5 μF/cm^2^ from the simplified LGK model. 

In the GK model, the cell body is characterized as basal and apical membranes stacked together, and the transcellular currents passing through both membranes are the same [3]. With the assumption of C_a_ = C_b_, the specific impedance of the stacked cell membranes is the impedance of the two identical cell membranes connected in series. However, the apical cell membrane usually has more foldings than the basal cell membrane, indicating a higher apical membrane capacitance than the basal membrane capacitance (C_a_ > C_b_). In the modified GK model, we consider different current distributions through the basal and apical membranes and successfully estimate C_a_ and C_b_ separately. As shown in Table 2, C_a_ and C_b_ values obtained from the modified GK model are 2.7 μF/cm^2^ and 2.1 μF/cm^2^ for HaCaT cells and 4.2 μF/cm^2^ and 2.8 μF/cm^2^ for HUVEC cells. The analyzed data shown in Table 2 also display that the parameter R_b_ obtained from the simplified LGK model or the modified GK model has a similar value, and so does the parameter α. This result indicates the effectiveness of applying the modified GK model (a two-path model) for the impedance analysis of cells with low junctional resistances, such as HaCaT and HUVEC cells.

## 5. Conclusions

In this paper, we have developed and validated simplified LGK and modified GK models for the impedance analysis of epithelial and endothelial cells measured by ECIS. Our results provide model parameter and cell characteristic values by computing the specific impedance of the cell-covered electrode (Zc) and fitting the experimental data. Because of the sensitivity of the system and the straightforwardness of the numerical calculation, the impedance analysis of cell layers measured by ECIS will find more applications in investigating cell behaviors in tissue culture. The cell–electrode models developed here can be used for future modeling and measuring studies of epithelial and endothelial cells.

## Figures and Tables

**Table 1:** Impedance analysis of MDCK and OVCA429 cells using LGK, simplified LGK, and modified GK models.

	R_b_ (Ω·cm^2^)	α (Ω^1/2^·cm)	C_a_ (μF/cm^2^)	C_b_ (μF/cm^2^)	C*_l_* (μF/cm^2^)
MDCK (LGK)	52.9 ± 4.9	22.1 ± 0.9	4.6 ± 0.2	2.9 ± 0.2	1.2 ± 0.1
MDCK (simplified LGK)	54.1 ± 4.9	22.1 ± 0.9	4.7 ± 0.2	2.8 ± 0.2	1.5 ± 0.2
MDCK (modified GK)	61.7 ± 5.4	20.0 ± 0.9	4.5 ± 0.2	3.6 ± 0.2	
OVCA429 (LGK)	87.3 ± 7.4	115.4 ± 9.3	4.4 ± 0.2	2.6 ± 0.1	1.1 ± 0.1
OVCA429 (simplified LGK)	84.5 ± 6.9	115.1 ± 9.3	4.5 ± 0.3	2.6 ± 0.1	0.9 ± 0.1
OVCA429 (modified GK)	109.0 ± 7.1	102.4 ± 7.8	3.7 ± 0.2	2.9 ± 0.1	

Values are means ± standard error; *n* = 14 for MDCK cells; and *n* = 16 for OVCA429 cells. For the simplified LGK model, we used Equations (18)–(20) and five parameters, R_b_, α, C_a_, C_b_, and C*_l_*, to fit the measured resistance and capacitance spectra of cell-covered electrodes. For the modified GK model, we used Equations (29) and (30) and four parameters, R_b_, α, C_a_, and C_b_, to fit the measured impedance spectra. See the list of symbols for the definition of parameters. Percentage errors are less than 15% for LGK and simplified LGK model fitting and less than 10% for modified GK model fitting.

**Table 2:** Impedance analysis of HaCaT and HUVEC cells using simplified LGK and modified GK models.

	R_b_ (Ω·cm^2^)	α (Ω^1/2^·cm)	C_a_ (μF/cm^2^)	C_b_ (μF/cm^2^)	C*_l_* (μF/cm^2^)
HaCaT (simplified LGK)	1.4 ± 0.3	3.6 ± 0.2	2.9 ± 0.2	1.6 ± 0.2	1.7 ± 0.4
HaCaT (modified GK)	1.6 ± 0.3	3.4 ± 0.2	2.7 ± 0.1	2.1 ± 0.1	
HUVEC (simplified LGK)	0.6 ± 0.2	5.0 ± 0.1	3.5 ± 0.2	2.5 ± 0.3	1.0 ± 0.2
HUVEC (modified GK)	0.7 ± 0.2	4.9 ± 0.1	4.2 ± 0.3	2.8 ± 0.2	

Values are means ± standard error; *n* = 4 for HaCaT cells; and *n* = 16 for HUVEC cells. Percentage errors are less than 15% for simplified LGK model fitting and less than 10% for modified GK model fitting.

## Data Availability

The data presented in this study are available upon reasonable request from the corresponding author.

## Glossary

*Z_c_* (Ω·cm^2^)	specific impedance of the cell-covered electrode
*Z_n_* (Ω·cm^2^)	specific impedance of the cell-free electrode
*Z_a_* (Ω·cm^2^)	specific impedance through the apical cell membrane
*Z_b_* (Ω·cm^2^)	specific impedance through the basal cell membrane
Z*_l_* (Ω·cm^2^)	specific impedance through the lateral cell membrane
*C_a_* (μF/cm^2^)	specific capacitance of the apical cell membrane
*C_b_* (μF/cm^2^)	specific capacitance of the basal cell membrane
C*_l_* (μF/cm^2^)	specific capacitance of the lateral cell membrane
*V* (volt)	electrical potential in the space between the cell layer and electrode
*V_c_* (volt)	applied electrical potential across the cell
*V_i_* (volt)	electrical potential inside the cell
*I* (A)	electrical current through the space between cells layer and electrode
*I_c_* (A)	total current across the cell-covered electrode
*I_ct_* (A)	total current from the area of a single cell
*I_i_* (A)	transcellular current through the apical cell surface
*I_l_* (A)	paracellular current through the lateral cell membrane
*R_b_** (Ω)	junctional resistance between adjacent cells
*R_b_* (Ω·cm^2^)	junctional resistance between adjacent cells over a unit cell area
*r_c_* (μm)	cell radius
*ρ* (Ω·cm)	resistivity of the cell culture medium
*h* (nm)	average height between the basal cell surface and the substratum
*l* (μm)	length of the lateral intercellular space
